# Balancing Functionality and Safety in Food Packaging Coatings

**DOI:** 10.3390/foods15030571

**Published:** 2026-02-05

**Authors:** Athina Ntzimani, Theofania Tsironi

**Affiliations:** 1 Laboratory of Chemistry, Analysis & Design of Food Processes, Department of Food Science and Technology, University of West Attica, Egaleo Park Campus, 28 Ag. Spyridonos Str., 122 43 Egaleo, Greece; 2 Laboratory of Food Process Engineering, Department of Food Science and Human Nutrition, Agricultural University of Athens, 75 Iera Odos Str., 118 55 Athens, Greece

**Keywords:** functional coatings, bio-based films, recycled coatings, nanomaterials, migration, regulation, safe-by-design

## Abstract

Functional coatings for food packaging offer innovative approaches to extend shelf life, preserve quality and introduce active properties such as antimicrobial or antioxidant effects. These coatings include natural bio-based films (e.g., polysaccharide or protein-based) and synthetic polymers enhanced with additives or nanomaterials. Despite their advantages (e.g., improved barrier properties, spoilage inhibition, or intelligent sensing) they also pose safety concerns. Migration of chemical constituents and additives into food can lead to toxicological risks, such as cytotoxicity or endocrine disruption. Non-intentionally added substances (NIASs) and nano-sized components further complicate safety assessments. This review outlines the main types of functional coatings, their active mechanisms, and associated safety issues. Particular focus is placed on migration phenomena, chemical interactions and health risks from common migrants including plasticizers, monomers, nanoparticles and essential oils. The EU Packaging and Packaging Waste Regulation (Regulation (EU) 2025/40), adopted in December 2024 and published in the Official Journal in January 2025, introduces comprehensive sustainability and substance-restriction requirements, including strict criteria for food packaging materials that will apply from 12 August 2026. Emerging challenges include the assessment of bio-based and recycled coatings and the toxicology of nanomaterials. Balancing functionality with safety remains crucial for next-generation, sustainable and health-compliant food packaging.

## 1. Introduction

Food packaging plays a critical role not only in containing and protecting foods but also in preserving quality and extending shelf life. In recent years, functional coatings have emerged as an innovative approach to enhance packaging performance by imparting active or intelligent properties, such as improved oxygen barriers properties, antimicrobial activity, or real-time sensing capabilities, beyond the traditional passive protection offered by conventional materials. These developments reflect growing consumer and industry demands for longer shelf life, reduced food waste, and enhanced food safety and quality. Among the leading innovations are natural biopolymer coatings (e.g., edible films based on polysaccharides, proteins or lipids) and synthetic polymer coatings that incorporate active additives. In addition to the base polymer matrix, the performance of food packaging coatings is often enhanced through the incorporation of structural modifiers such as fillers and plasticizers. Reinforcing fillers, including modified cellulose (e.g., nanocellulose or cellulose derivatives) and modified hydroxyapatite, have gained attention for improving the mechanical strength, barrier properties, and thermal stability of bio-based and synthetic films. These materials can also influence moisture sensitivity and structural integrity, which are critical for maintaining coating functionality during storage. Plasticizers are another essential component, particularly in biopolymer-based films, where they reduce brittleness and enhance flexibility. Common plasticizers such as glycerol, sorbitol, polyethylene glycol, and citrate esters are widely used, although their presence may also affect diffusion processes and migration behavior, linking material design directly to safety considerations. Such systems aim to create packaging that interacts with the food or its environment to maintain freshness, for example by scavenging oxygen or releasing antimicrobial agents [1].

However, the introduction of new materials and additives into food packaging raises important safety concerns. Food contact materials (FCMs) are required not to transfer their constituents to food in amounts that could endanger human health or alter food composition or sensory properties. In practice, many packaging components can migrate into food to varying extents [2]. These migrants may include intentionally added substances (IASs), which are designed to impart functionality, such as preservatives, antioxidants, or nanoparticles, as well as non-intentionally added substances (NIASs), which arise as impurities or degradation products during manufacturing and use. For instance, low-molecular-weight compounds from polymers, plasticizers, inks, or adhesives can leach into foods under certain conditions, introducing potentially hazardous chemicals into the food chain. High-profile cases, such as bisphenol A (BPA) from can linings and phthalate plasticizers from plastics, have heightened public concern due to their endocrine-disrupting effects. These examples underscore the central challenge addressed in this review: achieving an optimal balance between enhanced functionality and consumer safety in modern food packaging coatings [3].

This review provides an updated and comprehensive analysis of this balance. We first survey the current landscape of functional coatings in food packaging, spanning both natural and synthetic systems and their main applications. The subsequent section explores active and intelligent mechanisms, including antimicrobial, antioxidant, and barrier functionalities. Safety and migration issues are also examined, focusing on the transfer of coating constituents (including nanoparticles) into foods and associated toxicological risks, such as cytotoxicity and genotoxicity. The EU Packaging and Packaging Waste Regulation (Regulation (EU) 2025/40), adopted in December 2024 and published in the Official Journal in January 2025, introduces comprehensive sustainability and substance-restriction requirements, including strict criteria for food packaging materials that will apply from 12 August 2026. Finally, strategies to reconcile functionality and safety are discussed, through safer-by-design materials, controlled-release systems, and advanced toxicological testing, and future challenges are identified, such as NIAS identification, nano-additive safety, and the risk assessment of bio-based and recycled packaging. In contrast to previous reviews that emphasize either performance or toxicology in isolation, this work highlights the interplay between functionality and consumer safety, with particular attention to NIAS, nanomaterials, and recent European regulatory developments. This review is intended for researchers and professionals of food packaging, materials science, and food safety, offering guidance for the design of coatings that combine technological effectiveness with regulatory compliance and health protection.

## 2. Functional Coatings in Food Packaging: Current Landscape

Functional coatings are thin surface layers or treatments applied to packaging materials, or directly to food products in the case of edible coatings, to impart specific functional or enhanced properties. Unlike conventional inert packaging, which acts primarily as a passive barrier, functional coatings are intentionally designed to interact beneficially with the food or its surrounding environment [4]. These coatings can be classified into four main categories: (i) Barrier coatings, which improve resistance to gases, moisture, or oils; (ii) active coatings, which provide antimicrobial, antifungal, or antioxidant protection; (iii) intelligent or smart coatings, capable of sensing, monitoring or indicating changes (e.g., through color shifts); and (iv) sustainable coatings, designed to enhance compostability, recyclability, or to replace environmentally harmful materials, such as plastics or PFAS-based layers.

### 2.1. Natural vs. Synthetic Coatings

A fundamental distinction in the current research landscape lies between natural, bio-based coatings and synthetic polymer-based coatings. Natural coatings often take the form of edible films applied directly to foods such as fruits, vegetables, and cheeses or used as biodegradable wrapping materials. They are formulated from renewable biopolymers including polysaccharides (e.g., starch, cellulose, chitosan, alginate, pectin), proteins (gelatin, casein, soy protein), and lipids (waxes, fatty acid composites), frequently blended with resins or gums to improve mechanical and barrier properties [5,6]. To impart bioactive functionality, natural additives such as plant extracts and essential oils are commonly incorporated, imparting antimicrobial or antioxidant functions. These coatings are typically edible or compostable and closely align with consumer demand for clean-label and sustainable packaging solutions. Consequently, bio-based coatings derived from renewable resources have become a major focus of recent research [7,8]. Numerous studies have demonstrated their effectiveness in preserving perishable food; for instance, chitosan or alginate coatings enriched with essential oils have been shown to inhibit surface microbial growth on produce or meat, thereby significantly extending shelf life while remaining safe for consumption [9].

In contrast, synthetic functional coatings are typically based on conventional packaging polymers, such as polyethylene (PE), polypropylene (PP), polyethylene terephthalate (PET), or polylactic acid (PLA), which are coated or co-extruded with additional layers containing active additives. Examples include oxygen-barrier coatings (e.g., thin layers of polyvinyl alcohol or ethylene vinyl alcohol), moisture or oil barriers on paperboard (formerly based on petroleum waxes or fluoropolymers) and UV-protective coatings containing UV absorbers like benzophenones or nano-titanium dioxide [10]. Many functional coatings are specifically engineered as carriers for active agents; for example, antimicrobial films may incorporate biocidal layers containing silver or zinc oxide nanoparticles, or immobilize natural antimicrobials such as bacteriocins or bacteriophages to suppress microbial growth at the food surface [4,11,12]. Inorganic coatings, including nanoscale layers of SiO_2_ or Al_2_O_3_ deposited on plastic or glass, constitute another important class of high-barrier systems already implemented at an industrial scale. These ultrathin layers (typically < 100 nm) drastically reduce oxygen and moisture transmission without compromising transparency or weight, thereby enhancing food stability and extending shelf life [13].

### 2.2. Commercial Applications and Emerging Examples

Several functional coatings have successfully transitioned from research to commercial application. Edible coatings are already applied to various types of fresh produce (e.g., wax or resin coatings on apples and citrus) to reduce moisture loss and microbial spoilage, representing some of the earliest and most widely adopted examples of functional coatings. Chitosan-based antimicrobial coatings have shown considerable promise in extending the freshness of meats and fish [14]. In plastic packaging, active films incorporating ethanol-releasing sachets or surface coatings are used for bakery products to effectively inhibit mold growth. Synthetic barrier coatings, such as polyvinylidene chloride (PVDC) or acrylic-based coatings, have long been employed to enhance gas barrier properties in multilayer films. However, increasing environmental and health concerns are driving the gradual replacement of these legacy materials: PVDC, due to its chlorine content, poses disposal challenges, while fluoropolymer coatings on paper are being phased out due to the persistence and toxicity of per- and polyfluoroalkyl substances (PFASs), which are expected to be banned in the EU by 2026 [15,16].

An emerging frontier involves smart coatings that integrate sensing or color-indicating functionalities. Recent reviews emphasize that large-scale deployment of intelligent/smart packaging is still constrained by added unit cost, sensor/indicator stability and reliability under real cold-chain conditions, and the lack of harmonized validation/standardization for performance claims. Commercial uptake is further limited by manufacturing integration challenges (e.g., printing/lamination compatibility and robustness during high-speed converting), and by regulatory/safety considerations for indicator chemistries and potential migrants. A recent study provides a comprehensive overview of sensor and indicator systems developed for real-time quality monitoring, while also evaluating barriers to adoption such as cost, sensor stability under cold-chain conditions, and the lack of regulatory harmonization—directly relevant to understanding practical constraints in smart coatings [17]. Other studies detail scalable fabrication strategies for food quality sensors and discuss material integration issues that affect both performance and manufacturability [18]. A study by Park and Kwon (2026) examine the sustainability aspects and lifecycle considerations of smart packaging technologies, including environmental trade-offs associated with embedded electronics and printed components [19]. Abekoon et al. (2024) extend this perspective by evaluating synergies between intelligent packaging design, AI-driven data interpretation, and the needs of dynamic food supply chains, emphasizing real-world implementation challenges [20]. Although these technologies are not yet widely commercialized, research has demonstrated intelligent coatings capable of changing color in response to temperature abuse (time–temperature indicators) or to pH variations associated with food spoilage, effectively transforming packaging into a freshness indicator [18,21,22]. These systems typically employ halochromic dyes or redox-sensitive compounds embedded within a polymer matrix. Ensuring the safety of these intelligent additives is therefore critical; to prevent contamination, they are usually positioned on the external surface of packaging or physically separated from the food by an internal barrier layer [23].

## 3. Functional Additives and Mechanisms of Action

The performance of functional food packaging coatings depends largely on the type of active additives incorporated and the mechanisms by which they operate. These additives can be naturally derived compounds, such as plant extracts, enzymes, or biopolymers, or synthetic agents, including engineered polymers, inorganic particles, or man-made antimicrobials. This section outlines the major classes of functional additives, their mechanisms of action, and considerations regarding their efficacy and safety as summarized in Table 1 [24].

### 3.1. Antimicrobial Agents and Mechanisms

A key objective of active packaging is to inhibit the growth of spoilage or pathogenic microorganisms on food surfaces. Antimicrobial additives are therefore among the most extensively studied and applied functional agents in coating systems [25].

#### 3.1.1. Natural Antimicrobials

These include essential oils (EOs) and their active constituents (e.g., thymol from thyme, eugenol from clove, cinnamaldehyde from cinnamon), plant extracts rich in phenolic compounds, organic acids (e.g., sorbic or lactic acid), enzymes such as lysozyme, and bacteriocins such as nisin [26]. Natural antimicrobials are attractive due to their broad-spectrum activity, Generally Recognized as Safe (GRAS) status, and consumer-friendly perception. Their mechanisms of action typically involve disruption of microbial cell membranes, denaturation of proteins, or interference with metabolic processes. For example, the lipophilic nature of essential oil components allows them to partition into bacterial membranes, compromising structural integrity and causing leakage of cellular contents, ultimately leading to cell death [9]. Similarly, bacteriocins such as nisin form pores in the membranes of Gram-positive bacteria, leading to cell depolarization and death [27]. Many natural antimicrobials also exhibit antioxidant properties, providing dual functionality for food preservation [28,29].

#### 3.1.2. Mechanisms of Release

In packaging coatings, natural antimicrobials may be immobilized on the surface (providing contact-active antimicrobial effects) or intentionally released through diffusion or volatilization. Volatile EOs commonly act through headspace release—for instance, vapors of oregano oil inhibiting mold growth on bakery products—while non-volatile compounds such as nisin or lysozyme are generally immobilized or dispersed within the coating matrix and require direct contact with the food surface. The activity of many natural antimicrobial and antioxidant agents is closely linked to their chemical functional groups. Phenolic compounds and essential oil constituents, such as thymol or eugenol, contain phenolic hydroxyl (–OH) groups and aromatic structures that enable hydrogen donation, radical scavenging, and interactions with lipid membranes, leading to membrane destabilization [30]. Organic acids exert antimicrobial effects primarily through their carboxyl (–COOH) groups, which facilitate acidification and diffusion of the un-dissociated form across microbial membranes [31]. Similarly, chitosan’s antimicrobial activity is associated with protonated amino (–NH_3_^+^) groups that interact electrostatically with negatively charged microbial cell surfaces, disrupting membrane integrity [32]. These structure–activity relationships explain the dual functional and biological performance of many coating additives. The release rate is critical: too rapid a release can result in sensory defects and premature depletion, whereas overly slow release may lead to insufficient microbial inhibition. To address this, encapsulation techniques (e.g., nanoencapsulation in cyclodextrins or liposomes) have been developed to control release kinetics and protect sensitive compounds from volatilization or degradation. For instance, encapsulating thyme oil in chitosan nanoparticles within an edible film has been shown to provide gradual, sustained antimicrobial activity and improved stability during storage [32].

#### 3.1.3. Synthetic Antimicrobials

Conventional approaches once employed synthetic biocides such as triclosan or quaternary ammonium compounds in food-contact materials; however, regulatory and toxicity concerns have largely curtailed their use. More recent strategies employ polymeric antimicrobials—polymers with intrinsic antimicrobial activity (e.g., polyhexamethylene biguanide)—or surface-modified materials that disrupt microbial cells upon contact through electrostatic or hydrophobic interactions. Another class includes photoactive coatings incorporating photocatalysts such as TiO_2_, which generate reactive oxygen species under UV illumination to inactivate surface microbes. Although effective, such systems rely on external activation (e.g., light exposure) and raise potential safety concerns regarding radical formation [33].

#### 3.1.4. Metal-Based Antimicrobials

Among inorganic options, silver nanoparticles (AgNPs) have received extensive attention and limited commercial adoption due to their potent and broad-spectrum antimicrobial properties. Silver ions (Ag^+^) released from AgNPs interact with microbial proteins and DNA, disrupt enzymatic activity, and induce oxidative stress. Similarly, zinc oxide (ZnO) and copper oxide (CuO) nanoparticles exert antimicrobial activity through combined mechanisms of ion release, membrane damage, and the generation of reactive oxygen species. The performance of these materials depends on their incorporation and stabilization within the coating matrix: effective antimicrobial action can be achieved through controlled ion release while minimizing particle migration into food. Metal-based nanoparticles show differences in antimicrobial specificity: AgNPs exhibit broad-spectrum activity against both Gram-negative and Gram-positive bacteria through Ag^+^ interactions with thiol groups in proteins and DNA, ZnO nanoparticles are often more effective against Gram-positive bacteria and fungi via reactive oxygen species generation, and CuO nanoparticles display broad antibacterial and antifungal activity associated with Cu^2+^ release and oxidative stress, with vegetative cells being generally more susceptible than spores [33,34,35]. For example, EFSA’s evaluation of silver nanoparticles embedded in a polymer matrix demonstrated negligible nanoparticle migration, with only ionic silver release observed within regulatory limits —achieving antimicrobial efficacy without compromising food safety [36].

#### 3.1.5. Bio-Based Antimicrobials from Fermentation

A growing area of interest involves antimicrobial agents derived from microbial fermentation, such as bacteriocins (e.g., pediocin, natamycin) and bacteriophages. Packaging films incorporating bacteriophages have been developed to selectively target pathogens like Listeria monocytogenes on ready-to-eat meats, offering highly specific antimicrobial action without affecting beneficial microflora [37]. Bacteriophages act by binding to bacterial cells, injecting their genetic material, and lysing the target bacteria [12]. Their localized activity, typically confined to the package–food interface, makes them promising tools for surface-targeted microbial control [38].

### 3.2. Antioxidants and Oxygen Scavengers

Oxidative reactions, such as lipid rancidity, vitamin degradation, and enzymatic or non-enzymatic browning, are major contributors to food quality loss. Packaging coatings may incorporate antioxidants that delay oxidation processes and maintain sensory and nutritional quality during storage.

#### 3.2.1. Natural Antioxidants

Compounds like tocopherols (Vitamin E), ascorbic acid (Vitamin C), rosemary extract (carnosic acid), green tea polyphenols (catechins), and many flavonoids serve to scavenge free radicals and reactive oxygen, thereby protecting the food. The natural antioxidants incorporated into functional coatings are predominantly of plant origin and belong mainly to phenolic compounds and antioxidant vitamins. Key sources include aromatic herbs and spices such as rosemary and oregano, which are rich in phenolic diterpenes (e.g., carnosic acid, carnosol) and phenolic acids; tea leaves, such as green tea, which provide catechins; fruits and vegetables that supply ascorbic acid and diverse flavonoids; and oilseeds and vegetable oils that are natural reservoirs of tocopherols (vitamin E). These compounds are widely studied in active packaging systems because their radical-scavenging and metal-chelating mechanisms can retard lipid oxidation while maintaining a food-grade origin and favorable consumer perception [28,39]. These can be either released into the food or act at the interface. For example, a biodegradable film with green tea extract can slowly release catechins into a food matrix (like ground meat) to retard lipid oxidation, or an LDPE film with embedded tocopherol might bloom tocopherol to the surface and then into the food fat over time. Antioxidants usually work by donating hydrogen atoms to quench free radicals in the food, stopping the chain reactions of lipid peroxidation [39].

#### 3.2.2. Enzymatic Oxygen Scavengers

Glucose oxidase + catalase systems have been studied, where glucose oxidase (in the presence of glucose, which can be included in a coating) converts O_2_ and glucose into gluconic acid and hydrogen peroxide, and then catalase breaks down the hydrogen peroxide. This effectively removes oxygen in a sealed package. These enzymes can be immobilized in an edible coating sachet or coating on an inside layer of a package. The mechanism is biochemical and continues until the substrate (glucose) is exhausted, thereby extending the protective effect [40].

#### 3.2.3. UV Absorbers and Light Stabilizers

While not antioxidants per se, UV-blocking additives (like titanium dioxide or UV-absorbing chemicals) prevent light from catalyzing oxidation reactions in foods (for instance, light-induced oxidation of riboflavin in milk). By shielding the food from UV, they indirectly act as preservers of quality [41]. UV-absorbing additives used in food packaging coatings include inorganic particles such as titanium dioxide (TiO_2_) and zinc oxide (ZnO), as well as organic UV absorbers like benzophenones and benzotriazole derivatives. These are mainly applied in packaging for light-sensitive products—such as milk, edible oils, fatty foods, and beverages—to prevent photo-oxidation, vitamin degradation, and off-flavor development. However, disadvantages include the possible migration of low-molecular-weight organic UV stabilizers, the potential endocrine activity of certain benzophenones, opacity or whitening effects (for inorganic particles), and the risk that photodegradation of the polymer matrix under UV exposure may generate additional non-intentionally added substances (NIASs) [41,42,43].

### 3.3. Intelligent and Responsive Additives

For coatings with intelligent functions (indicators, sensors), the additives used have mechanisms tied to environmental triggers: (1) Thermochromic pigments might contain leuco dyes and developers that change structure (hence color) at certain temperatures. For example, some are based on a crystal violet lactone system that is colorless when complexed with a developer at low temperature but separates and becomes colored at higher temperature. These can be in a polymer coating on the package and indicate temperature abuse by an irreversible color change [44]. (2) pH-sensitive dyes, mainly anthocyanins incorporated into bio-based films, may exhibit color changes in response to pH (e.g., red in acid, purple in neutral, green/yellow in alkaline). In a spoilage scenario, as meat spoils, it releases amines raising pH; an anthocyanin-based indicator film might change colors, signaling spoilage. The mechanism is the well-known structural change in the anthocyanin chromophore in response to pH (flavylium cation vs. quinoidal base forms) [21]. (3) Gas sensors, developed with dyes or nanoparticles that react with specific gases (e.g., H_2_S produced by fish spoilage), are another mechanism. One example is that a film containing lead acetate will turn black in the presence of H_2_S (forming lead sulfide); obviously, lead is not food-safe, but similar mechanisms with safer chemicals are under exploration. Palladium or gold nanoparticles can change optical properties when they absorb hydrogen—these principles are being examined for intelligent packaging, albeit mostly being in the research phase [45,46].

### 3.4. Digestibility in the Context of Functional and Safe Food Coatings

In the context of balancing functionality and safety in food packaging coatings, digestibility is an important consideration when coatings are intended for direct consumption with food. Several edible coatings are formulated from biopolymers such as polysaccharides, proteins and lipids, which are generally regarded as safe and exhibit predictable gastrointestinal behavior. However, enhancing coating performance, such as improving mechanical strength, barrier properties or antimicrobial activity, may involve chemical modifications, crosslinking, or the incorporation of active agents that can influence digestibility. Current research aims to optimize coating functionality while preserving physiological safety by using food-grade, enzymatically degradable materials and minimizing non-digestible or bioaccumulative components. Limitations remain, particularly regarding the potential impact of high crosslinking density or functional additives on gastrointestinal degradation and bioaccessibility. Therefore, digestibility represents a key safety parameter that must be considered alongside functional performance to ensure that advanced food packaging coatings remain both effective and safe for consumers [28,29,39,47].

## 4. Mechanisms and Safety Considerations

The mechanisms that enable functional performance in coatings—such as controlled release, interfacial activity, and barrier modulation—are intrinsically linked to safety, because the same physicochemical processes that determine efficacy also govern the migration, exposure, and toxicological relevance of active and non-intentionally added substances. While the above mechanisms focus on functionality, it is important to note that the efficacy and the safety of these additives are often intertwined. For instance, controlled release is a mechanism to achieve prolonged antimicrobial or antioxidant action, but from a safety perspective, it also means controlling the amount that ends up in the food [48]. In functional coatings, the mechanisms governing the release of active substances—such as diffusion through the polymer matrix, volatilization, or triggered release from encapsulated systems—represent a critical interface between performance and safety. Parameters including polymer structure, additive solubility, temperature, and food composition simultaneously influence antimicrobial or antioxidant efficacy and determine consumer exposure, meaning that release kinetics act as a central design variable linking functionality with toxicological risk. Encapsulation not only modulates release but can prevent a sharp burst of a compound that might exceed flavor thresholds or safety limits. Many natural antimicrobials (like essential oils) have strong flavors, so their mechanism of gradual release is as much about maintaining sensory acceptance as it is about sustaining antimicrobial activity. In other cases, an additive might work well functionally but pose toxicological issues if it migrates too freely (e.g., some UV stabilizers like benzophenone derivatives can migrate and have been found to be endocrine-active; hence, newer developments look at photostable coatings that immobilize such stabilizers or use polymer-bound alternatives) [42]. Functional additives in food packaging coatings operate through diverse mechanisms. These include killing or inhibiting microorganisms (via chemical or physical means), scavenging oxygen or free radicals, forming physical barriers that limit the transmission of deteriorative agents, and responding to environmental cues to provide information. Each additive’s mechanism underpins its benefits and also must be considered in light of material compatibility and regulatory limits. These mechanistic considerations therefore form the basis for understanding migration phenomena and toxicological risk, which are examined in detail in the following section.

## 5. Safety Concerns and Migration Issues

While functional coatings offer significant benefits for food preservation and quality, they also introduce safety concerns primarily related to the potential migration of chemicals from the coating into the food. The term migration refers to the transfer of molecules from the packaging material to the food, driven by concentration gradients and diffusion processes, and sometimes facilitated by the food matrix (especially if the food is fatty or has solvents that can extract packaging components). Safety concerns arise when migrated substances have toxicological profiles that could harm consumers, or when they adversely affect the food’s composition and organoleptic properties. Key issues include the presence of known toxic migrants (like monomers, additives, heavy metals), the unknown effects of NIAS, the unique behavior of nanomaterials, and the cumulative or cocktail effects of multiple migrants [49]. In active packaging systems, migration is often an intrinsic outcome of the intended release mechanism rather than an accidental process. Consequently, safety assessment must consider not only total migration but also release rate, duration of exposure, and food–material interactions, as these factors govern whether active substances remain within regulatory and toxicological limits while still delivering technological benefits.

### 5.1. Overall Migration and Specific Migration

Migration from food packaging coatings may occur both through the coating or film matrix itself and through individual components incorporated within the film. In conventional coatings, migration is typically unintentional and involves low-molecular-weight substances such as residual monomers, additives, degradation products, or non-intentionally added substances (NIASs) diffusing into food. In contrast, functional and active coatings are often designed to enable intentional migration, for example through the controlled release of antimicrobial or antioxidant agents. Consequently, safety assessment of food packaging coatings must consider migration arising from both the film as a whole and its individual constituents, taking into account the intended functionality, release mechanisms, and regulatory requirements. Regulators often distinguish between overall migration (the total mass of all substances that migrate from a material into food simulants, typically with a general limit such as 10 mg of total migrants per dm^2^ of packaging in the EU) and the specific migration of particular chemicals (which may have individual limits). Exceeding overall migration limits indicates a material is not suitably inert. Functional coatings, by design, may intentionally release substances (active packaging), so they blur the line between inert packaging and additive release. For active packaging, regulations (like EU Regulation 450/2009) require that any intentionally released substances comply with food safety regulations as if they were direct food additives. For example, if a coating releases an antioxidant into food, that antioxidant must be an approved food additive or a food-grade substance with established safety for use in foods (e.g., substances with GRAS status in the U.S. or authorized food additives under EU legislation), when used in compliance with food contact material regulations and used within acceptable limits. Specific migration is a major concern for certain chemicals, as will be explored in further detail below. 

#### 5.1.1. Monomers and Oligomers

Residual monomers from polymers (e.g., styrene from polystyrene, vinyl chloride from PVC, caprolactam from nylon, bisphenol A from polycarbonate or epoxy linings) can migrate. BPA, in particular, has been a high-profile case—used in epoxy can coatings, it can leach into foods and has been linked to endocrine disruption. This led to bans in baby bottles and a new stringent tolerable daily intake proposed by EFSA’s 2023 re-evaluation, which reduced the BPA tolerable daily intake from the 2015 temporary TDI of 4 µg/kg bw/day to 0.2 ng/kg bw/day (i.e., 20,000-fold lower), essentially suggesting that even very low BPA exposures could be of concern. As a result, industry has moved to BPA-free can coatings (though replacements like bisphenol S are also under scrutiny for similar effects) [50]. Oligomers (short-chain fragments of polymers) often fall under NIAS and can migrate as well; some recent studies identified polyester oligomers migrating from PET coatings on cans, the safety of which is not fully characterized.

#### 5.1.2. Plasticizers and Additives

Phthalate plasticizers (like DEHP, DINP) used in some plastics are known to migrate into fatty foods and are classified as reproductive toxins and endocrine disruptors. They have largely been restricted or replaced by alternative plasticizers in food packaging. Similarly, additives like antioxidants in plastics (e.g., Irganox 1010, Irgafos 168) can degrade into smaller NIAS such as 2,4-di-tert-butylphenol, which have been found in food simulant extracts. Kato and Conte-Junior [43] noted that degradation products of such antioxidants are a primary source of NIAS in plastics, implying that even additives added for the stability of the plastic can form migratable substances. These degradation products can have toxicological effects; for instance, some are suspected endocrine disruptors or irritants.

#### 5.1.3. Photoinitiators and Ink Components

A well-known incident in 2005 involved ITX (isopropylthioxanthone), a photoinitiator from printing ink on carton packages, migrating into baby milk and causing a recall [51]. This highlighted the risk from printing inks and adhesives on packaging, which can migrate especially if there is set-off (where printed outer layer substances transfer to the food contact side when rolled or stacked). Many ink components (benzophenone photoinitiators, amines, etc.) are not intended to contact food but can if packaging is improperly designed. These substances can impart off-flavors and also pose health risks (benzophenone was found to be toxicologically concerning and is now restricted for food packaging uses in the EU). This underscores the importance of barriers—e.g., using functional barrier coatings that prevent ink chemicals from reaching food [3].

#### 5.1.4. Per- and Polyfluoroalkyl Substances (PFASs)

Used as grease-proofing agents on paper packaging (fast-food wrappers, bakery papers), PFASs are extremely persistent chemicals and have been linked to cancer, immune system harm, and other issues. The EU Packaging and Packaging Waste Regulation (Regulation (EU) 2025/40), adopted in December 2024 and applicable from 12 August 2026, introduces strict limits on per- and polyfluoroalkyl substances (PFASs) in food contact packaging under Article 5. From that date, packaging containing PFASs above specified concentrations (e.g., 25 ppb for individual PFAS and 250 ppb for total targeted PFAS) may not be placed on the EU market, effectively prohibiting intentional use of PFASs in these materials. This regulatory restriction reflects the precautionary approach to persistent fluorinated substances in the food contact context and aligns with broader EU policy objectives for sustainable packaging design [52].

### 5.2. Non-Intentionally Added Substances (NIASs) and Complex Mixtures

NIASs represent one of the most challenging aspects of packaging safety. By definition, NIASs are chemical species present in a material but not added on purpose; they include impurities from raw materials, by-products of reactions, oligomers, breakdown products, and contaminants [43]. In functional coatings, which often have complex formulations (multiple components, novel materials), NIASs can be numerous. Analytical studies using techniques like GC-MS and LC-QTOF have revealed that a given packaging film can contain hundreds of detectable chemicals, many of which are NIAS and unidentified. For example, a recent analysis of bio-based polymer coatings found NIASs stemming from natural resin acids and terpenes used in formulation, which migrated in low amounts [53].

The safety concern with NIAS is that their toxicity is often unknown—standard regulatory approvals focus on known additives. EU regulations (10/2011) explicitly state that NIASs must be assessed for risk by the manufacturer using scientific principles, and EFSA’s 2016 Scientific Opinion provided a framework: if an NIAS is identified and quantifiable, one should assess its toxicity; if it is unidentified, one may apply the Threshold of Toxicological Concern (TTC) concept to gauge risk. The TTC provides conservative intake thresholds (based on structural class of the chemical) below which the risk of toxicity is considered negligible. Often, a generic 0.01 mg/kg food threshold is used for unknown NIASs (assuming worst-case, Cramer Class III), meaning that if an NIAS migrates in an amount of less than 10 ppb into food, it is unlikely to be a safety concern unless it is a known genotoxic carcinogen. If it migrates in an amount above that, or if the structure suggests potential genotoxicity (e.g., it contains structural alerts), dedicated toxicological testing is warranted. Kato and Conte-Junior [43] emphasize that bioassays combined with high-resolution analytical methods are an effective approach to prioritizing NIASs—for instance, an in vitro cytotoxicity or mutagenicity test on a total migrate can flag if any NIASs could be hazardous, guiding further analysis.

Functional coatings that incorporate recycled or natural materials might introduce unique NIASs. For instance, if a coating is made from a plant extract, it could contain a variety of secondary metabolites; most may be harmless at low levels, but some could be allergens or have pharmacological activity [2].

### 5.3. Nanomaterials: Migration and Toxicological Implications

Nanomaterials (NMs) used in packaging coatings—such as metal nanoparticles, nanoclays, and nanocellulose—require particular attention due to potential safety considerations [54]. Key concerns relate to their possible migration from packaging materials into food, either as intact particles or as dissolved species, and the potential toxicological risks associated with the ingestion of these materials or their released ions. Migration of nanoparticles is generally understood based on the following: large particles above a certain size have very limited migration due to the sieve-like barrier of polymer matrices. Many nanoparticles, although tiny (1–100 nm), tend to form aggregates in polymers—effectively making their migratable size larger. EFSA’s evaluation of silver nanoparticles (mean size ~15 nm) in plastic noted that the particles largely stayed embedded and did not migrate as particles. Only silver in ionic form (Ag^+^) was detected in food simulants at low levels (a few µg/ kg). Recent comprehensive reviews highlight the complexity of risk assessment for nanomaterials in food contact applications, including multi-scale migration mechanisms, bioabsorption and toxicological pathways, and tailored regulatory risk assessment principles that incorporate physico-chemical characterization and exposure paradigms. This is in line with other studies: nano-silica or nano-titania in plastics also show minimal particulate migration, with any migration mostly in dissolved form or as very small agglomerates [55]. However, certain conditions can increase nanoparticle release: high temperatures, acidic or alcoholic foods (which might dissolve or dislodge particles), or mechanical stresses could in theory release nanoparticles. Studies on polymer nanocomposites have shown that well-embedded nanoclays do not significantly migrate, but poorly exfoliated ones might flake off microscale fragments. Each case is material-specific, so risk assessment of nanomaterials often requires data on particle size distribution in simulants after contact [56].

### 5.4. Cytotoxicity and In Vitro Testing

Researchers frequently employ in vitro cytotoxicity assays to screen packaging-film extracts for potential hazards before application. For example, a recent study evaluated extracts from a carbohydrate-based biodegradable film—incorporating polysaccharides, proteins, and algal bioactive components—for cytotoxic effects on human intestinal (Caco-2) cells, and found no significant reduction in cell viability, indicating that the migrants present in the film were likely of low toxicological concern [57]. In the study by Kalliampakou et al. [47], an edible packaging film based on carboxymethyl cellulose and sodium alginate was subjected to migration testing with food simulants followed by in vitro assays on human intestinal and liver cell lines, and the authors found that migrants did not reduce cell viability nor induce oxidative stress, supporting its potential safety for food contact applications. While in vitro tests are not full toxicological evaluations, they are useful for flagging potential issues early. They also help in investigating NIASs: for instance, if a packaging migrant mixture shows positive in vitro genotoxicity, it signals the need for deeper analysis to find the culprit compounds.

## 6. Regulatory Framework and Risk Assessment

Ensuring the safety of food packaging coatings is a shared responsibility between industry and regulators. Over the years, a robust regulatory framework has been established in many jurisdictions, aiming to preemptively control which substances can be used in food contact materials (FCMs) and under what conditions. The cornerstone of EU food contact material regulation is Regulation (EC) No 1935/2004, often called the Framework Regulation. Article 3 establishes the fundamental safety requirements: materials and articles in contact with food must not release components into food at levels that endanger human health or that cause an unacceptable change in the food’s composition, taste, or odor. This applies to all food contact materials, including coatings. In practice, this is a general provision and is complemented by more specific measures for certain material types.

Good Manufacturing Practice (GMP) is mandated by Regulation (EC) No 2023/2006, ensuring that packaging is produced in a way that minimizes contaminants and inconsistencies. For coatings, GMP might cover things like the proper curing of can coatings to minimize monomer residuals, or using purified additives. Adherence to GMP is particularly relevant for controlling NIASs (e.g., preventing contamination, ensuring complete reactions to avoid leftover reagents).

The EU has detailed specific regulations for some materials.

### 6.1. Plastics Regulation (EU) No 10/2011

This regulation is highly relevant because many coatings (especially synthetic ones) are polymeric and fall under “plastic” or are multi-materials containing plastic layers. It provides a Union List of authorized substances (monomers, additives) that can be used in plastic FCMs, each with restrictions like specific migration limits (SMLs) if needed. For example, an additive used in a plastic coating must be on this list (unless it is a polymer production aid, colorant, or a non-intentionally added substance, as these are treated differently). If a company develops a new functional polymer coating for food packaging that involves a novel monomer or additive not on the list, they must submit a dossier to EFSA for evaluation.

### 6.2. Active and Intelligent Packaging Regulation (EC) No 450/2009

Recognizing that active packaging (which releases substances) and intelligent packaging (which might have indicators) do not fit neatly into inert packaging rules, the EU set specific provisions. It requires that active components that are intended to be released meet food safety requirements as if they were direct food additives or ingredients. Such components either have to be already authorized as food additives/flavorings or go through a safety evaluation for their use via packaging, as for example with a coating that releases an antimicrobial like lysozyme—lysozyme is a permitted enzyme in some foods, but its use via packaging still requires that it does not exceed levels that would be unsafe or alter the food beyond regulatory limits. The regulation also requires appropriate labeling: active packaging must indicate it is active (so consumers do not accidentally consume sachets or think something is wrong if, say, an oxygen absorber sachet is present), and any instructions to not consume certain parts if applicable.

Intelligent packaging must likewise not have components (like a dye in an indicator) that migrate into food in unsafe amounts, and any information provided (like spoilage indicator color) should not be misleading. While there is not a list of authorized indicator substances, they would be subject to Article 3 of 1935/2004—so safety is determined by case-specific assessment.

The EU Packaging and Packaging Waste Regulation (Regulation (EU) 2025/40), adopted in December 2024 and published in the Official Journal in January 2025, introduces comprehensive sustainability and substance restriction requirements, including strict criteria for food packaging materials that will apply from 12 August 2026. The PPWR introduces binding requirements on recyclability, the use of recycled content, and the minimization of hazardous substances in packaging, explicitly affecting food contact coatings. It addresses problematic legacy chemicals, such as PFASs used in grease-resistant coatings, and signals their phase-out in food packaging, reflecting a precautionary approach where environmental persistence and human health concerns outweigh functional benefits. By integrating chemical safety, material circularity, and sustainability objectives, the PPWR complements existing food contact legislation (e.g., Regulation 1935/2004 and Regulation 10/2011) and is expected to drive innovation toward safer, bio-based, and recyclable coating technologies while maintaining high food safety standards.

## 7. Designing Safe-by-Design Functional Coatings for Food Packaging

The development of functional food packaging coatings necessitates a “safe-by-design” philosophy, in which technological performance is integrated from the outset with consumer safety, regulatory compliance, and sustainability considerations. Many promising functional coatings fail at the commercialization stage due to migration, toxicity, or regulatory barriers, highlighting the need for a holistic design strategy. This approach begins with the careful selection of inherently safer materials, such as food-grade substances with established safety for use in foods (e.g., substances with GRAS status in the U.S. or authorized food additives under EU legislation), when used in compliance with food contact material regulations and bio-based polymer matrices, combined with green chemistry principles that prioritize purity and the avoidance of hazardous auxiliaries or chemicals of concern. While natural materials often offer toxicological advantages, issues such as allergenicity, dose-dependent effects, and long-term stability must also be systematically addressed [2,52].

Controlled release strategies play a critical role in balancing functionality and safety, particularly for coatings designed to deliver active compounds. Encapsulation, triggered-release mechanisms, and surface-bound actives can significantly reduce consumer exposure while maintaining efficacy. By moderating release rates, activating only under specific conditions, or immobilizing active agents on the coating surface, these approaches limit unnecessary migration into food while still providing antimicrobial or antioxidant benefits. In parallel, the use of functional barrier layers (either within multilayer packaging structures or as thin topcoats in coating systems) can effectively limit the migration of substances from non-food-contact layers. Under EU legislation for plastics (Regulation (EU) No 10/2011), non-authorized substances may be used behind a functional barrier only under specific conditions: they must not be classified as CMR substances (unless explicitly authorized), must not be in nanoform unless permitted, and their migration into food must remain below 0.01 mg/kg food (10 ppb), while also complying with the general safety requirements of Regulation (EC) No 1935/2004 (no risk to human health, no unacceptable change in food composition or organoleptic properties). Thus, functional barriers are a risk management tool to restrict exposure, rather than a blanket authorization to use otherwise non-compliant substances [58].

The recent literature provides concrete ‘safe-by-design’ demonstrations: (i) For nano-enabled antimicrobial coatings, EFSA’s assessment of silver nanoparticles embedded in plastics concluded that migration of particles is negligible under intended use, with only low-level release of ionic silver detected—illustrating a design strategy where the functional agent is immobilized within the matrix to minimize consumer exposure while retaining surface activity. (ii) For bio-based active films, Kalliampakou et al. [47] proposed an integrated safe-by-design workflow that couples standardized migration testing with in vitro cytotoxicity screening of film migrates, enabling early reformulation (e.g., polymer/additive selection and dose optimization) before scale-up to ensure functionality without compromising safety.

Achieving this balance requires an iterative and interdisciplinary development process, combining functionality testing, early-stage migration screening, formulation optimization, and real-food validation. Close collaboration among materials scientists, food technologists, toxicologists, and regulatory experts allows potential safety and compliance issues to be identified early and addressed through design modifications rather than post hoc corrections. Ultimately, such an integrated strategy ensures that innovations in functional food packaging coatings deliver real performance benefits while aligning with public health objectives, regulatory expectations, and long-term sustainability goals [2].

From a sustainability and circular economy perspective, bio-based functional coatings (e.g., polysaccharides, proteins, plant-derived additives) offer advantages such as renewability, potential biodegradability or compostability, and alignment with reduced fossil-resource dependence. They can also enable valorization of agro-industrial by-products (e.g., pectin, polyphenol-rich extracts), contributing to resource efficiency. In contrast, synthetic polymer-based coatings often provide superior barrier performance and durability but may hinder recyclability when applied as multilayer or incompatible coatings, complicating material recovery streams. The transition toward circular packaging therefore relies on designing functional coatings that maintain performance while being compatible with recycling processes, minimizing hazardous additives, and supporting emerging regulatory goals for safe, recyclable, and bio-based materials.

## 8. Future Outlook: Harmonizing Safety and Innovation

The balance between functionality and safety in food packaging coatings is inherently dynamic, as emerging challenges continually reshape performance requirements. New foodborne pathogens, climate change-driven alterations in spoilage patterns, and evolving distribution systems increasingly demand more advanced packaging functionalities. Nevertheless, safety remains the non-negotiable parameter for any innovation. Looking ahead, the field is expected to rely more heavily on predictive and preventive design strategies that integrate safety considerations at the earliest stages of material development, rather than addressing them retrospectively during regulatory evaluation or commercialization [52].

Computational modeling and in silico tools are poised to play a central role in this transition. By predicting migration behavior and toxicological profiles from molecular structure, these approaches can enable chemists and material designers to optimize additives before synthesis, reducing potential exposure while preserving functional performance. In parallel, high-throughput screening methodologies, similar to those used in pharmaceutical development, offer the potential to rapidly assess large numbers of candidate materials for safety at an early stage [59,60].

Regulatory frameworks will also need to evolve in response to increasingly complex and innovative packaging technologies. More adaptive regulatory mechanisms may be required to accommodate demonstrably safe innovations without imposing prohibitive timelines or costs, particularly for smaller companies and startups. At the same time, greater transparency regarding the chemical composition of food packaging materials is likely to become a cornerstone of future safety management. Comprehensive databases of packaging-related chemicals and known migrants can support cumulative exposure assessments and guide the substitution of potentially problematic substances with safer alternatives.

Ultimately, balancing functionality and safety is a matter of systematic risk management, aimed at maximizing benefits such as improved food preservation, reduced waste, and enhanced consumer convenience while minimizing health risks and unintended consequences. In addition to direct consumer exposure, future research should also address the environmental fate of functional coating components, including the release and transformation of micro- and nanoscale materials during disposal, recycling, or degradation, which represents a similar but distinct risk-assessment challenge beyond the food contact framework. Past experience has shown that failures to adequately address safety, even unintentionally, can erode consumer trust and trigger stricter regulatory responses that may hinder the adoption of otherwise valuable technologies. Proactively embedding safety into the innovation process therefore serves both public health objectives and the long-term interests of industry. By consolidating recent evidence across materials science, food technology, toxicology, and regulation, this review identifies critical knowledge gaps and research priorities that must be addressed to ensure that future functional coatings deliver technological benefits without compromising consumer health or regulatory compliance.

## Figures and Tables

**Table 1:** Advantages and limitations of major classes of functional additives in food packaging coatings.

Additive Class	Typical Examples	Main Advantages	Key Limitations
Natural antimicrobials	Essential oils, phenolics, nisin, lysozyme	GRAS/food-grade status; broad antimicrobial spectrum; consumer-friendly; antioxidant activity in most cases	Strong odor/flavor; volatility and instability; variable efficacy; potential allergenicity; fast depletion
Synthetic antimicrobials	Quaternary ammonium, polymeric biocides, photoactive TiO_2_	High and predictable efficacy; durability; contact-active mechanisms	Regulatory restrictions; possible cytotoxicity; consumer perception issues; risk of resistance
Metal-based nanoparticles	AgNPs, ZnO, CuO	Potent broad-spectrum antimicrobial activity; thermal stability; long-term action	Public and regulatory concern; potential ion migration; accumulation risks; cost; nano-specific assessments needed
Natural antioxidants	Tocopherols, ascorbic acid, rosemary, green tea extracts	Food-grade; dual-antioxidant/antimicrobial effects; nutritional profile enhancement	Limited stability; rapid consumption; color/flavor impact; lower efficiency than synthetics
Oxygen scavengers (enzymatic)	Glucose oxidase/catalase	Highly effective O_2_ removal; targeted function; minimal sensory impact	Moisture dependence; limited lifetime; formulation complexity; enzyme stability
UV absorbers/light stabilizers	TiO_2_, benzophenones	Protection of light-sensitive foods; improvement of shelf life	Migration of organic UV absorbers; endocrine activity concerns; opacity issues
Intelligent dyes/pigments	Anthocyanins, leuco dyes, redox dyes	Real-time freshness/abuse indication; low material usage; added value	Stability issues; potential migration; interpretation by consumers; limited standardization
Bacteriophages/biologics	*Listeria* phages, natamycin	High specificity; minimal impact on microflora; bio-based	Narrow spectrum; regulatory complexity; stability during processing; public acceptance

## Data Availability

No new data were created or analyzed in this study. Data sharing is not applicable to this article.

## References

[1] Tsironi T.N., Jaiswal S., Bahmid N.A., Jaiswal A.K. (2026). Introduction to Food Packaging: Historical Overview, Sustainability, and Current Trends. Sustainable Food Packaging: Safety and Sustainability Aspects of Food Packaging.

[2] Geueke B., Groh K., Muncke J. (2018). Food Packaging in the Circular Economy: Overview of Chemical Safety Aspects for Commonly Used Materials. J. Clean. Prod..

[3] Muncke J. (2009). Exposure to Endocrine Disrupting Compounds via the Food Chain: Is Packaging a Relevant Source?. Sci. Total Environ..

[4] Duncan T.V. (2011). Applications of Nanotechnology in Food Packaging and Food Safety: Barrier Materials, Antimicrobials and Sensors. J. Colloid Interface Sci..

[5] Priyadarshi R., Rhim J.-W. (2020). Chitosan-Based Biodegradable Functional Films for Food Packaging Applications. Innov. Food Sci. Emerg. Technol..

[6] Rhim J.-W., Park H.-M., Ha C.-S. (2013). Bio-Nanocomposites for Food Packaging Applications. Prog. Polym. Sci..

[7] Tsokri S., Sarafidou M., Tsouko E., Athanasopoulou E., Vardaxi A., Pispas S., Tsironi T., Koutinas A. (2024). Efficient pectin recovery from sugar beet pulp as effective bio-based coating for Pacific white shrimp preservation. Int. J. Biol. Macromol..

[8] Sarafidou M., Tsouko E., Vardaxi A., Athanasopoulou E., Pispas S., Tsironi T., Koutinas A. (2025). Exploiting Electrospinning Technology with Sugar Beet Pulp-Derived Pectin Reinforced by Pullulan for Novel Fiber Materials. Carbohydr. Polym..

[9] Ribeiro-Santos R., Andrade M., Melo N.R., Sanches-Silva A. (2017). Use of Essential Oils in Active Food Packaging: Recent Advances and Future Trends. Trends Food Sci. Technol..

[10] Wang L., Zhao X., Yu J., He Y. (2021). Functional Polymer Coatings for Food Packaging Applications: Barrier Properties, Active Additives, and Safety Considerations. Compr. Rev. Food Sci. Food Saf..

[11] Bahrami A., Delshadi R., Mahdi Jafari S., Williams L. (2019). Nanoencapsulated nisin: An engineered natural antimicrobial system for the food industry. Trends Food Sci. Technol..

[12] Stafyli C.A., Athanasopoulou E., Tseperka M., Skliros D., Tsironi T., Flemetakis E. (2026). Integration of a Marine Bacteriophage into Chitosan, Sodium Alginate and Methylcellulose-Based Coatings to Control Microbial Spoilage and *Vibrio alginolyticus* Growth in Fish. Int. J. Food Microbiol..

[13] Tavakolian S., Ahari H., Givianrad M.H., Hosseini H. (2021). Improving the Barrier Properties of Food Packaging by Al_2_O_3_@TiO_2_ and Al_2_O_3_@SiO_2_ Nanoparticles. Food Bioprocess. Technol..

[14] Giatrakou V., Ntzimani A., Savvaidis I.N. (2010). Combined Chitosan–Thyme Treatments with Modified Atmosphere Packaging on a Ready-to-Cook Poultry Product. J. Food Prot..

[15] Ribes Ortega C., Molitorisová A., Purnhagen K. (2025). Dangerous Legacy of Food Contact Materials on the EU Market: Recall of Products Containing PFAS. Eur. J. Risk Regul..

[16] Gupta R.K., Pipliya S., Karunanithi S., Eswaran U G.M., Kumar S., Mandliya S., Srivastav P.P., Suthar T., Shaikh A.M., Harsányi E. (2024). Migration of Chemical Compounds from Packaging Materials into Packaged Foods: Interaction, Mechanism, Assessment, and Regulations. Foods.

[17] Davidescu M.A., Pânzaru C., Mădescu B.M., Poroșnicu I., Simeanu C., Usturoi A., Matei M., Doliș M.G. (2025). Advances and Challenges in Smart Packaging Technologies for the Food Industry: Trends, Applications, and Sustainability Considerations. Foods.

[18] Mkhari T., Adeyemi J.O., Fawole O.A. (2025). Recent Advances in the Fabrication of Intelligent Packaging for Food Preservation: A Review. Processes.

[19] Park E., Kwon K. (2026). Smart and sustainable food packaging: Recent advances in active/intelligent technologies and future directions. Scifood.

[20] Abekoon T., Buthpitiya B.L.S.K., Sajindra H., Samarakoon E.R.J., Jayakody J.A.D.C.A., Kantamaneni K., Rathnayake U. (2024). A comprehensive review to evaluate the synergy of intelligent food packaging with modern food technology and artificial intelligence field. Discov. Sustain..

[21] Basdeki E., Maurizzi E., Bigi F., Quartieri A., Pulvirenti A., Tsironi T. (2025). Functionality and storage evaluation of fish freshness indicators based on the incorporation of anthocyanins extracted from winery grape pomace into polyvinyl alcohol/starch films. Packag. Technol. Sci..

[22] Zhai X., Xue Y., Sun Y., Ma X., Ban W., Marappan G., Tahir H.E., Huang X., Wu K., Chen Z. (2025). Colorimetric Food Freshness Indicators for Intelligent Packaging: Progress, Shortcomings, and Promising Solutions. Foods.

[23] Azeredo H.M.C., Correa D.S. (2021). Smart choices: Mechanisms of intelligent food packaging. Curr. Res. Food Sci..

[24] Upadhyay P., Sazar S., Sharma M., Verma R., Boparai K.S., Sharma A. (2024). A Review on Advanced Antimicrobial Food Packaging: Natural and Synthetic Additives, Mechanisms and Safety Aspects. Polymers.

[25] Fadiji T., Rashvand M., Daramola M.O., Iwarere S.A. (2023). A Review on Antimicrobial Packaging for Extending the Shelf Life of Food. Processes.

[26] Ntzimani A.G., Giatrakou V.I., Savvaidis I.N. (2011). Combined natural antimicrobial treatments on a ready-to-eat poultry product stored at 4 and 8 °C. Poultry Sci..

[27] Popa E.E., Miteluț A.C., Râpă M., Popescu P.A., Drăghici M.C., Geicu-Cristea M., Popa M.E. (2022). Antimicrobial Active Packaging Containing Nisin for Preservation of Products of Animal Origin: An Overview. Foods.

[28] Choulitoudi E., Ganiari S., Tsironi T., Ntzimani A., Tsimogiannis D., Taoukis P., Oreopoulou V. (2017). Edible coating enriched with rosemary extracts to enhance oxidative and microbial stability of smoked eel fillets. Food Packag. Shelf Life..

[29] Ntzimani A., Kalamaras A., Tsironi T., Taoukis P. (2023). Shelf Life Extension of Chicken Cuts Packed under Modified Atmospheres and Edible Antimicrobial Coatings. Appl. Sci..

[30] Burt S. (2004). Essential oils: Their antibacterial properties and potential applications in foods—A review. Int. J. Food Microbiol..

[31] Theron M.M., Lues J.F.R. (2011). Organic Acids and Food Preservation.

[32] Hosseinnejad M., Jafari S.M. (2016). Evaluation of Different Factors Affecting Antimicrobial Properties of Chitosan. Int. J. Biol. Macromol..

[33] Díez-Pascual A.M. (2020). Antimicrobial Polymer-Based Materials for Food Packaging Applications. Polymers.

[34] Lemire J.A., Harrison J.J., Turner R.J. (2013). Antimicrobial activity of metals: Mechanisms, molecular targets and applications. Nat. Rev. Microbiol..

[35] Sirelkhatim A., Mahmud S., Seeni A., Kaus N.H.M., Ann L.C., Bakhori S.K.M., Hasan H., Mohamad D. (2015). Review on zinc oxide nanoparticles: Antibacterial activity and toxicity mechanism. Nano-Micro Lett..

[36] European Food Safety Authority (EFSA) CEP Panel (2021). Safety assessment of the substance silver nanoparticles for use in food contact materials. EFSA J..

[37] López de Dicastillo C., Settier-Ramírez L., Gavara R., Hernández-Muñoz P., López Carballo G. (2021). Development of Biodegradable Films Loaded with Phages with Antilisterial Properties. Polymers.

[38] Wagh R.V., Priyadarshi R., Rhim J.-W. (2023). Novel Bacteriophage-Based Food Packaging: An Innovative Food Safety Approach. Coatings.

[39] Singh A.K., Kim J.Y., Lee Y.S. (2022). Phenolic Compounds in Active Packaging and Edible Films/Coatings: Natural Bioactive Molecules and Novel Packaging Ingredients. Molecules.

[40] Bazzoli P., Iametti S., Fessas D., Bonomi F., Schiraldi A. (2023). Oxidases as Oxygen Scavengers in Hypoxic Conditions: A Kinetic Model. Molecules.

[41] Tripathi S., Kumar L., Deshmukh R., Gaikwad K. (2024). Ultraviolet Blocking Films for Food Packaging Applications. Food Bioprocess. Technol..

[42] Chen M.-L., Chen C.-H., Huang Y.-F., Chen H.-C., Chang J.-W. (2022). Cumulative Dietary Risk Assessment of Benzophenone-Type Photoinitiators from Packaged Foodstuffs. Foods.

[43] Kato L.S., Conte-Junior C.A. (2021). Safety of Plastic Food Packaging: The Challenges about Non-Intentionally Added Substances (NIAS) Discovery, Identification and Risk Assessment. Polymers.

[44] Jamnicki Hanzer S., Kulčar R., Vukoje M., Marošević Dolovski A. (2023). Assessment of Thermochromic Packaging Prints’ Resistance to UV Radiation and Various Chemical Agents. Polymers.

[45] Heo W., Lim S. (2024). A Review on Gas Indicators and Sensors for Smart Food Packaging. Foods.

[46] Pierre L., Bruna Bugueño J.E., Leyton Bongiorno P.A., Torres Mediano A., Rodríguez-Mercado F.J. (2024). Colorimetric Indicator Based on Gold Nanoparticles and Sodium Alginate for Monitoring Fish Spoilage. Polymers.

[47] Kalliampakou K.I., Athanasopoulou E., Spanou A., Flemetakis E., Tsironi T. (2025). In vitro cytotoxicity evaluation of a CMC-SA edible packaging film for migration and safety assessment. Sci. Rep..

[48] Hou T., Ma S., Wang F., Wang L. (2023). A comprehensive review of intelligent controlled release antimicrobial packaging in food preservation. Food Sci. Biotechnol..

[49] Seref N., Cufaoglu G. (2025). Food Packaging and Chemical Migration: A Food Safety Perspective. J. Food Sci..

[50] European Food Safety Authority (EFSA) CEP Panel (2023). Re-Evaluation of the Risks to Public Health Related to the Presence of Bisphenol A (BPA) in Foodstuffs. EFSA J..

[51] European Food Safety Authority (EFSA) (2005). Opinion of the Scientific Panel on Food Additives, Flavourings, Processing Aids and Materials in Contact with Food on a Request from the Commission Related to Isopropylthioxanthone (ITX) in Food. EFSA J..

[52] Muncke J., Andersson A.-M., Backhaus T., Boucher J., Almroth B., Castillo A., Chevrier J., Demeneix B., Emmanuel J., Fini J.B. (2020). Impacts of Food Contact Chemicals on Human Health: A Consensus Statement. Environ. Health.

[53] Martínez-Bueno M.J., Hernando M.D., Uclés S., Rajski L., Cimmino S., Fernández-Alba A.R. (2017). Identification of Non-Intentionally Added Substances in Food Packaging Nano Films by Gas and Liquid Chromatography Coupled to Orbitrap Mass Spectrometry. Talanta.

[54] Muthu A., Nguyen D.H.H., Neji C., Törős G., Ferroudj A., Atieh R., Prokisch J., El-Ramady H., Béni Á. (2025). Nanomaterials for Smart and Sustainable Food Packaging: Nano-Sensing Mechanisms, and Regulatory Perspectives. Foods.

[55] Onyeaka H., Passaretti P., Miri T., Al-Sharify Z.T. (2022). The Safety of Nanomaterials in Food Production and Packaging. Curr. Res. Food Sci..

[56] Azeredo H.M.C. (2009). Nanocomposites for Food Packaging Applications. Food Res. Int..

[57] Martins V.F.R., Lopes A.I., Machado M., Costa E.M., Ribeiro T.B., Poças F., Pintado M., Morais R.M.S.C., Morais A.M.M.B. (2025). Biodegradable Films with Polysaccharides, Proteins, and Bioactive Compounds from *Lobosphaera* sp.: Antioxidant and Antimicrobial Activities. Foods.

[58] Zhang J., Zhang J., Zhang L., Qin Z., Wang T. (2025). Review of Recent Advances in Intelligent and Antibacterial Packaging for Meat Quality and Safety. Foods.

[59] Luijten M., Sprong R.C., Rorije E., van der Ven L.T.M. (2022). Prioritization of Chemicals in Food for Risk Assessment by Integrating Exposure Estimates and New Approach Methodologies: A Next-Generation Risk Assessment Case Study. Front. Toxicol..

[60] Turley A.E., Isaacs K.K., Wetmore B.A., Karmaus A.L., Embry M.R., Krishan M. (2019). Incorporating New Approach Methodologies in Toxicity Testing and Exposure Assessment for Tiered Risk Assessment Using the RISK21 Approach: Case Studies on Food Contact Chemicals. Food Chem. Toxicol..

